# Shifting Grounds—Facilitating Self-Care in Testing for Sexually Transmitted Infections Through the Use of Self-Test Technology: Qualitative Study

**DOI:** 10.2196/55705

**Published:** 2024-08-14

**Authors:** Bettina Trettin, Mette Maria Skjøth, Nadja Trier Munk, Tine Vestergaard, Charlotte Nielsen

**Affiliations:** 1 Department of Dermatology and Allergy Centre Odense University Hospital Odense Denmark; 2 Clinical Institute, Health Sciences University of Southern Denmark Odense Denmark; 3 Centre for Innovative Medical Technology Odense University Hospital Odense Denmark; 4 Department of Plastic Surgery Odense University Hospital Odense Denmark; 5 Department of Oral and Maxillofacial Surgery Odense University Hospital Odense Denmark

**Keywords:** chlamydia, sexually transmitted diseases, participatory design, self-test, qualitative, Chlamydia trachomatis, lymphogranuloma venereum, participatory, STD, STDs, sexually transmitted, sexually transmitted illness, sexually transmitted illnesses, STI, STIs, participatory, participation, self-testing, screening, health screening, asymptomatic screening, testing uptake

## Abstract

**Background:**

Chlamydia remains prevalent worldwide and is considered a global public health problem. However, testing rates among young sexually active people remain low. Effective clinical management relies on screening asymptomatic patients. However, attending face-to-face consultations of testing for sexually transmitted infections is associated with stigmatization and anxiety. Self-testing technology (STT) allows patients to test themselves for chlamydia and gonorrhea without the presence of health care professionals. This may result in wider access to testing and increase testing uptake. Therefore, the sexual health clinic at Odense University Hospital has designed and developed a technology that allows patients to get tested at the clinic through self-collected sampling without a face-to-face consultation.

**Objective:**

This study aimed to (1) pilot-test STT used in clinical practice and (2) investigate the experiences of patients who have completed a self-test for chlamydia and gonorrhea.

**Methods:**

The study was conducted as a qualitative study inspired by the methodology of participatory design. Ethnographic methods were applied in the feasibility study and the data analyzed were inspired by the action research spiral in iterative processes using steps, such as plan, act, observe, and reflect. The qualitative evaluation study used semistructured interviews and data were analyzed using a qualitative 3-level analytical model.

**Results:**

The findings from the feasibility study, such as lack of signposting and adequate information, led to the final modifications of the self-test technology and made it possible to implement it in clinical practice. The qualitative evaluation study found that self-testing was seen as more appealing than testing at a face-to-face consultation because it was an easy solution that both saved time and allowed for the freedom to plan the visit independently. Security was experienced when the instructions balanced between being detail-oriented while also being simple and illustrative. The anonymity and discretion contributed to preserving privacy and removed the fear of an awkward conversation or being judged by health care professionals thus leading to the reduction of intrusive feelings.

**Conclusions:**

Accessible health care services are crucial in preventing and reducing the impact of sexually transmitted infections and STT may have the potential to increase testing uptake as it takes into account some of the barriers that exist. The pilot test and evaluation have resulted in a fully functioning implementation of STT in clinical practice.

## Introduction

### Background

Chlamydia remains prevalent worldwide and is considered a global public health problem. However, testing rates among young sexually active people remain low. The majority of infected individuals are asymptomatic and potentially constitute a significant reservoir for transmission. In Denmark, far fewer men are tested than women despite men having the highest positivity rate in all age groups [1]. From 2018 to 2021, there was an increase in the positivity rate, and the largest increase was observed in 15- to 19-year-olds, where the positivity rate in 2021 was 36% for men and 26% for women. Remarkably, considerably fewer individuals were tested in 2020; however, the positivity rate was significantly higher than in 2019 [1]. This progression is worth taking seriously because untreated chlamydia can lead to complications, such as pelvic inflammatory disease and, in the worst-case scenario, ectopic pregnancies and infertility [2,3]. Thus, there is an urgent need to develop new ways to increase the testing uptake. In Denmark, general practitioners offer free testing and perform the majority of testing. Furthermore, 6 sexual health clinics in the country perform testing and screening for sexually transmitted infections (STIs). All of these testing options require that patients book an appointment and attend a face-to-face consultation, which may be a barrier for some patients because feelings of embarrassment and stigma are well-known deterrents to STI testing [4,5]. Young people, in particular, demand an alternative way of testing, with no explanation needed and minimal contact with health care professionals (HCPs) [6]. In Denmark, some municipalities offer home testing kits that can be ordered on the internet. Home tests are particularly popular among young people as they are perceived as easy and anonymous. However, the turnaround time for these tests is 10 days, plus delivery time, which is a challenge, as short waiting times are considered essential among young people who desire quick access to testing that can be integrated with school or work routines. Drop-in clinics are therefore popular and effective for detecting STIs at an early stage [7]. This knowledge has to be considered when developing new ways to increase testing uptake. In Denmark, testing uptake did not increase significantly despite national educational campaigns and programs by the Danish Health Authority. Thus, new innovative approaches are needed to reach the target group, and digital technologies may have the potential to support testing accessibility and meet challenges such as a lack of staff and emotional barriers linked to testing [8]. Therefore, we have designed and developed a self-testing technology (STT) that allows patients to be tested at a sexual health clinic through self-collected sampling without a face-to-face consultation, with no need to schedule an appointment. Instead, patients can use drop-in and visit sexual health clinics whenever they prefer.

### Objective

This study aimed to (1) pilot-test STT used in clinical practice and (2) investigate the experiences of patients who have completed a self-test for chlamydia and gonorrhea.

## Methods

### Study Design

The research was conducted as a qualitative study inspired by participatory design (PD) methodology. In health science, PD is often conducted in three phases, which include (1) identification of needs, (2) design and development, and (3) test and evaluation [9]. Genuine participation is considered essential, and the co-design in PD has the potential to design and develop future technologies based on users’ needs and adaptable to clinical practice. PD is characterized as a democratic research methodology in which mutual understanding emerges when all end users are involved in the change process [10]. Everyone affected by the technology gets a democratic voice and has a say and is therefore involved in its design. In this study, phase 1 consisted of literature studies, and the STT was designed based on research findings from several studies [6,11-14] that identified barriers in testing for STIs. Thus, the design and development of the STT was based on identified needs in the literature. In phase 2, a feasibility study was conducted to ensure the STT was feasible for clinical practice. It was considered an important step in the process because end users did not design the actual STT directly. However, the participant observations and structured interviews used to explore the patients’ experiences of using the STT were based on one of PDs core values: having a say and thus, giving them a voice to affect the outcome. In that way, the STT was co-designed, adjusted, and adapted based on end users’ experiences through the use of ethnographic methods. The further design and development phase was an iterative process that included end users and made necessary changes before implementation in clinical practice. In phase 3, a qualitative evaluation study was conducted to explore the users’ experiences of using the STT.

While PD inspired the overall study, 2 separate studies were conducted and analyzed: 1 feasibility study and 1 qualitative evaluation study, which were closely related. This paper will present the studies separately, although within the same methodological frame inspired by PD.

Four research group members were employed at the outpatient clinic; they consisted of nurses and 1 medical doctor. One was employed at another department. All members were experienced researchers; 4 have a PhD and 1 has an a masters in nursing science.

### Current Clinical Setting

The study was carried out at an outpatient clinic at a university hospital in Denmark, where patients can get free testing for STIs. A test requires a phone call to a secretary, who then will book the patient for a face-to-face consultation at the clinic within a day or two. During the consultation, HCPs obtain a medical record and ask questions about sexual (risk) behavior and symptoms. Patients will then be tested. To receive the test result, patients need to call a nurse trained in venereology.

### The Self-Testing Technology

**Figure 1:**
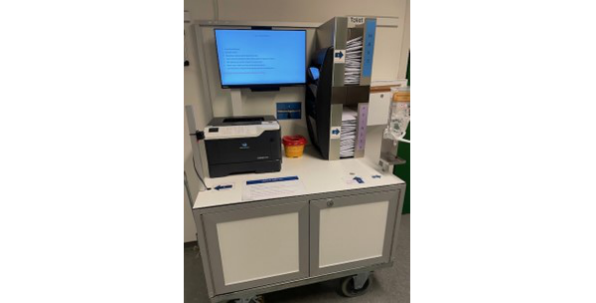
The self-testing technology.

During the COVID-19 pandemic, the university hospital placed several STTs on their property. HCPs used them for their mandatory COVID-19 throat swabs at the time. After the pandemic, the STTs were removed and no longer used. At the Department of Dermatology and Allergy Centre at the university hospital, the majority of patients tested for chlamydia and gonorrhea were young people with no symptoms who just wanted a check to be on the safe side. Having been introduced to the STTs, HCPs suggested using this technology to test and screen patients for STIs. Thus, an STT was rebuilt (Figure 1), and software was developed in close collaboration with the IT consultants that made its use possible for patients in clinical practice. The STT was placed at the outpatient clinic in a relatively quiet and undisturbed place. HCPs already trained in the field of venereology were introduced to the STT and the new workflow. The STT solution ensures anonymity and privacy in the way that users no longer need face-to-face consultation to test for chlamydia and gonorrhea. Instead, patients can use the drop-in facility and visit the sexual health clinic whenever they prefer. They will have to perform the test themselves using written instructions or video information.

Under the new system, patients who wanted to get tested for chlamydia and gonorrhea would call a nurse trained in the field of venereology, who would conduct a short interview for the patient’s medical records. The nurse would then set up the process in the electronic medical record that would give patients access to the STT using their personal identification number. Men were informed about having to self-collect a urine sample, and women about having to self-collect a vaginal swab. How to collect those samples was not elaborated because this information would be provided when patients used the STT. Patients were informed that they had 14 days to take the test within the opening hours of the outpatient drop-in clinic. Furthermore, they were informed about the location of the STT and that in case of a positive test result, they would receive an electronic letter in their secure personal digital mailbox. In case of a negative test, they would not be contacted but would have to check their test result on the Danish national portal for patient communication, a secure digital platform that contains all medical information linked to patients’ personal identification numbers. The unique personal identification numbers of all Danish citizens allow us to link medical information in different IT systems in a secure way. When entering the STT system the users will use their unique personal identification numbers and the system will recognize the user as a legitimate user of the system.

### Recruitment

For the feasibility study, asymptomatic patients who attended a face-to-face consultation were asked if they were willing to use the STT instead. If patients agreed and gave their oral consent, they were asked to fill out a written consent.

For the qualitative evaluation study, patients were recruited at the STT, where written consent forms were available. Patients who had filled out the consent forms were contacted by phone to schedule an interview. Patients were included using a purposive sampling strategy for both approaches to achieve diversity in sex, age, and geographical distance. All participants were older than 18 years, heterosexual, and were Danish-speaking.

### Data Collection

#### The Feasibility Study

Participant observation and informal interviews were carried out for the feasibility study. The participant observation aimed to gain insight into patient experience while following the instructions on how to find and use the STT. The participant observations were conducted based on American anthropologist James Spradley’s approach and thus concerned with a social situation [15]. An observational guide was developed based on Spradley’s 9 dimensions (Table 1) of a social situation to ensure that data were collected systematically and to provide structure to the observations in order not to miss important data. The social situation observed was patients using the STT for the first time. According to Spradley, a social situation concerns three elements, that are (1) a place, (2) actors, and (3) activities, and in order to understand this social situation, it first has to be described. Thus, making inferences makes it possible to discover meaning [15]. Therefore, in this study, inferences were made in relation to what the participants did (cultural behavior), the things they used (cultural artifacts), and what they said (cultural knowledge). The degree of participation can vary; however, passive participation was chosen in this study because the researchers might have influenced the outcome too much otherwise. Participants were asked to enter the front door, find their way to the STT, and take the test at the STT. Field notes were collected in a descriptive way to gain insight into possible obstacles and challenges while using the STT. After each session of participant observation, informal interviews with participants were conducted. Data were collected by the authors NTM and BT who are experienced in qualitative research. A structured interview guide was developed to obtain knowledge about the participants’ experiences using the STT. Participants were asked about the challenges, the information provided, suggestions for improvement, and their sense of security in using the STT. In total, 13 patients, 6 men and 7 women aged between 21 and 46 years were invited, and none declined to participate. During the participant observations, field notes were written, and informal interviews were recorded. All data were transcribed verbatim. Participants were recruited at the outpatient clinic and consisted of patients who had scheduled an appointment for a face-to-face consultation.

**Table 1:** Spradley’s 9 dimensions of a social situation.

Number	Dimensions	Place, actors, and activities
1	Space	The physical setting–location of the STT
2	Actor	Patients involved in the study–participants
3	Activity	Activities conducted by patients–using the STT
4	Object	Physical elements used by patients–the STT
5	Act	Individual actions taken by patients
6	Events	Context of the act–using the STT
7	Time	A sequence of events from beginning to end
8	Goal	What patients seek to accomplish–taking a self-test
9	Feeling	Emotions expressed by patients during the test

#### The Qualitative Evaluation

In total, 10 semistructured interviews were conducted with patients who had used the STT for the first time to explore their experiences and perceptions of the STT and, thereby, to gain insight into their experiences of having used it [16]. The interviews were carried out from October 2022 to January 2023 and were conducted at the location preferred by patients. An interview guide was developed to explore patients’ experiences, impressions, and acceptance of the STT. The interview guide was developed to ensure that participants could share their experiences and perceptions on using the STT, how they experienced the information provided, what, in their opinion, could be improved and why, how they experienced the access to STI testing in general, and wishes or requests they had for STI testing in the future. In total, 21 patients filled out a consent form, and 14 were contacted to schedule an interview. Of the 14 patients contacted, 1 did not show up for the interview, and 3 did not respond to our contact. In total, 10 patients aged between 18 and 32 years were included (6 females and 4 males). See Table 2 for participant characteristics. The interviews were conducted according to each participant’s preference, either at the sexual health clinic (n=2) or by phone (n=8). After conducting these interviews, the authors agreed that data saturation was reached and no further interviews needed to be conducted. The semistructured interviews were conducted by NTM, who is highly experienced in qualitative research. All transcripts were recorded and transcribed verbatim.

**Table 2:** Qualitative evaluation study.

Participant characteristics			Values
Median age (range), years			25 (18-32)
**Sex, n**			
	Male	4	
	Female	6	
**Employment status, n**			
	Employed	2	
	Student	8	
**Relationship, n**			
	Single	8	
	With partner	2	
**Previously tested, n**			
	Sexual health clinic	7	
	General practitioner	3	
	Checkpoint	2	
	No	1	

#### Ethical Considerations

The study was approved by the Danish Data Protection Agency (journal number 22/30101), following the principles of the Declaration of Helsinki [17]. All patients received verbal and written information about the studies and signed an informed consent form before data collection. For the qualitative evaluation study, participants received information about confidentiality and that only the person performing the interview would know their identity. They were ensured anonymity in both data analysis and reporting of the results. In order to respect the privacy of the potential participants for the qualitative study, patients themselves initiated the recruitment process. The authors fully acknowledged that participants during the interviews would elaborate on sensitive topics, therefore we chose not to recruit patients face-to-face while they were getting tested at the STT, thus, prioritizing patients’ interest and participating on a voluntary basis. Because patients were recruited during face-to-face consultations for the feasibility study, we did not collect other characteristics about the participants. Ethically, this seemed wrong since patients did not have time to think through whether they wanted to share more sensitive information with the researchers.

### Data Analysis

#### The Feasibility Study

The analysis of the feasibility study was inspired by the action research spiral in iterative processes [18] using the steps, that are plan, act, and reflect. The participant observation was conducted as a cyclical approach, where the reflected findings were shared with the research team before the next participant observation. Data analysis thus acted to adapt and modify the STT. Thus, each new activity and modification was based on shared reflections on the previous activity. These iterations were conducted until no further adjustments were required.

#### The Qualitative Evaluation

**Figure 2:**
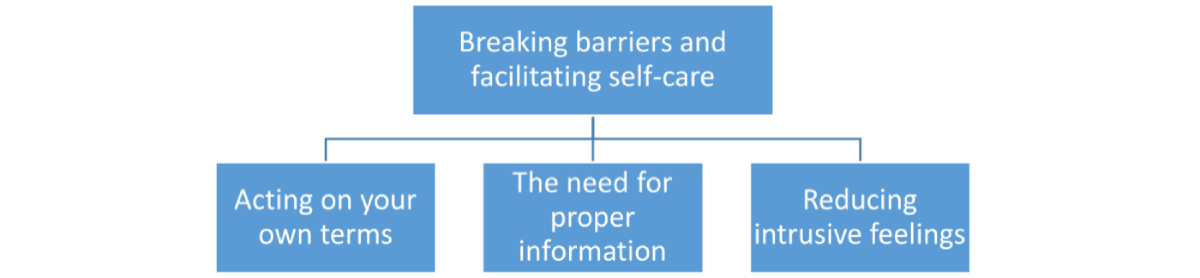
Results, main theme, and subthemes.

**Figure 3:**
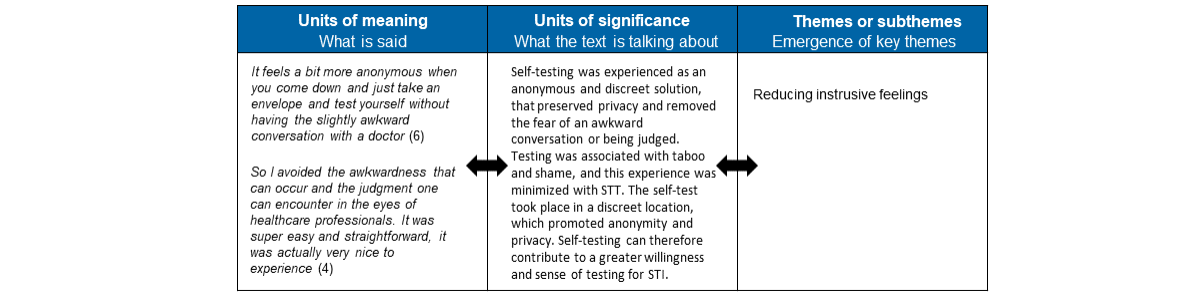
Example of the structural analysis.

The semistructured interviews, which aimed to explore experiences of the use of the STT, were analyzed inspired by Ricoeur’s theory of narrative and interpretation [19]. This is a 3-level analytical model that allows for interpretation of data collected through qualitative research methods such as semistructured interviews in order to gain insight into what patients experience [20]. This was carried out as a dialectical movement among three levels, which are (1) a naïve reading, (2) structural analysis, and (3) critical interpretation and discussion. First, all transcripts were gathered as one coherent text. Next, the transcripts were read and reread several times to get an initial impression of the text. This initial impression was the naïve reading and was written down. This step was performed by NTM. Then, a structural analysis was carried out where units of meaning (what the text said) and units of significance (what the text speaks about) were identified. Units of meaning were quotations from the data. Through a dialectical movement between understanding and explanation, by alternately distancing oneself from and coming closer to the text, a critical interpretation was possible and led to “units of significance.” This step was performed in collaboration through reflections and discussions to ensure saturation, agreement, and following the research objective and finally led to the identification of patterns, 1 main theme, and 3 subthemes. (Figure 2). All themes were subsequently interpreted and discussed in relation to theory and previous research results as part of the critical interpretation to gain an even deeper understanding. An example of the analysis is provided in Figure 3. The Consolidated Criteria for Reporting Qualitative Studies (COREQ) guided the reporting [21].

## Results

### Findings From the Feasibility Study

**Figure 4:**
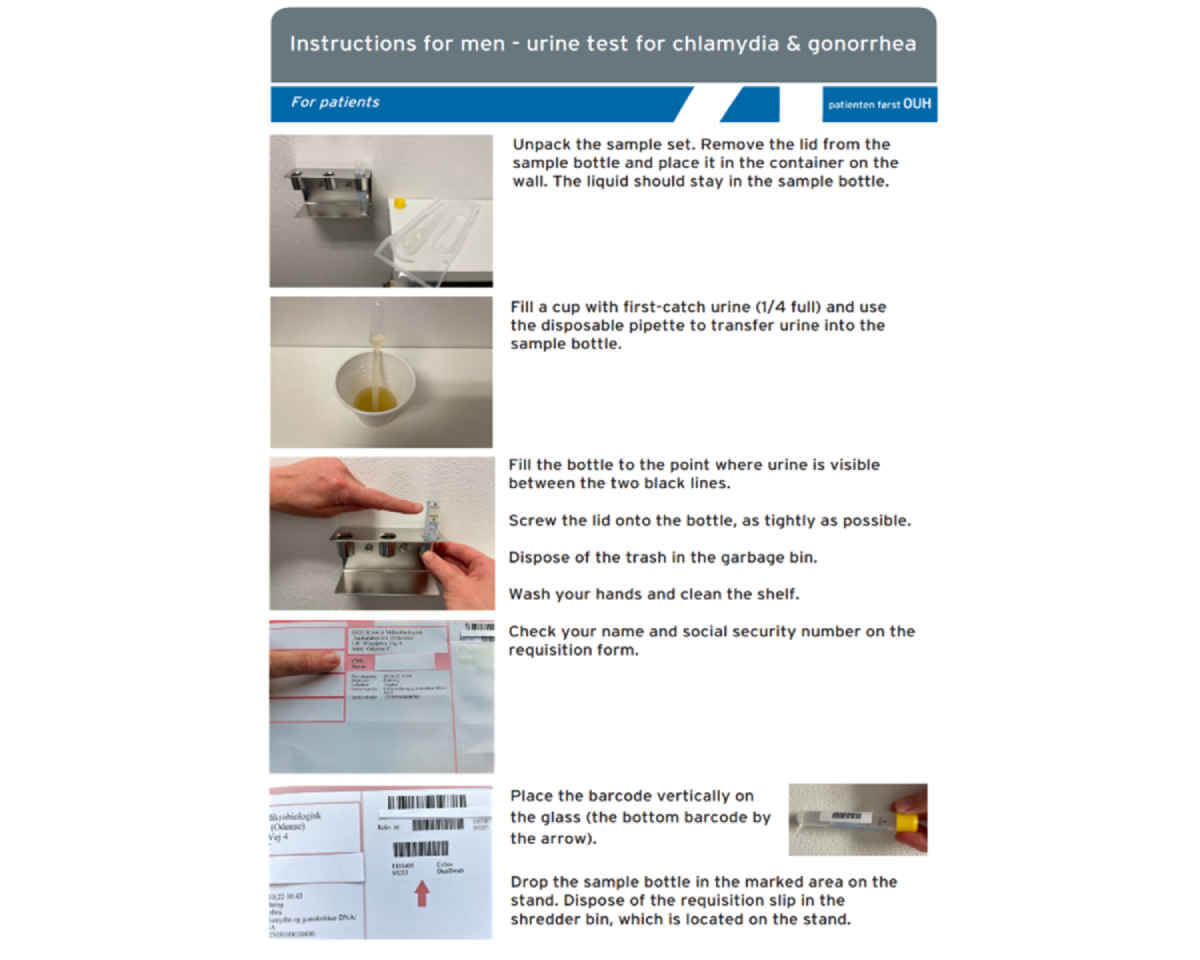
Example of revised instructions for male patients.

Patients had difficulties in finding the STT despite the signposting. Some had difficulties locating the STT because they had to enter through a door with a missing signpost. Others, following the blue arrows on the floor, entered the door but went straight into the toilet without looking up. Thus, the blue arrows on the floor were modified to point more directly at the STT. Furthermore, a signpost was added on the door patients had to enter. Patients had no problems scanning their social security cards; however, 2 patients could not print a requisition because of technical problems, and the HCPs had to print them. The reason for being unable to print the requisitions was technological, and the IT consultants analyzed these data and made the necessary changes at the STT. During the actual test, several problems occurred. Patients were insecure about which bar code to place on the sample, as the requisition had 2. Furthermore, they were not provided with sufficient information on correctly placing the bar code. Some patients were unsure whether the liquid inside the tests should be poured out. After the test, some patients did not know what to do with their used requisition. Thus, the written information for patients was adjusted and made extremely explicit (Figure 4). It was added to the written information that (1) the liquid should stay in the sample bottle; (2) an arrow along with text that clearly showed what bar code to place on the sample; and (3) a picture of how to place the bar code along with text. These findings led to the final modifications of the STT and made it possible to implement it in clinical practice. Thus, the users were directly involved in the design process based on participant observations and structured interviews. These user experiences collected through ethnographic methods facilitated co-design and made the STT adaptable to clinical practice.

### Findings From the Qualitative Evaluation

The naïve reading revealed that security was a central aspect for patients taking a test at the STT. Furthermore, it seemed that providing patients with the opportunity to take a test without having to face an HCP was experienced as a positive aspect.

### Breaking Barriers and Facilitating Self-Care

Breaking barriers were experienced on both a psychological and organizational level, creating feelings of security. This feeling of security was the central advantage in performing self-tests for STIs and was thus included in all 3 subthemes. Feelings of security facilitated self-care.

### Acting on One’s Own Terms

The flexibility and convenience of acting on one’s own terms were highlighted as significant factors in self-testing.

Convenience means something to many people. It’s incredibly easy to just make a phone call and order a test. It also matters that you don’t have a specific appointment time, but rather 2 weeks to get it done. I believe many people see this as an advantage. It makes it easy and manageable, so you get it done

9

Being able to take the test when it “fits in” and on one’s own terms could overcome barriers and give a sense of security and feelings of being independent of the system. For some, it made them more willing to get tested.

That was the easiest–I didn’t have to make an appointment with my own doctor. I could just decide for myself when I had a gap in my schedule to get it done

8

Self-testing was seen as more appealing than face-to-face testing because it was an easy solution that both saved time and allowed for the freedom to plan the visit independently. It was perceived as orderly and meaningful for its purpose, which could create motivation to self-care by getting tested, which increased the possibility of taking responsibility for one’s sexual health. On the other hand, the experience of hassle and meaninglessness of a standard face-to-face appointment could lead to hesitant behavior.

### The Need for Proper Information

The individual and illustrative instructions created a sense of security in performing the test correctly.

I found it to be very detailed and that it was very easy to follow the instructions and figure out what to do and in what order

9

Being taken by the hand and guided throughout the test was perceived as important. Some individuals experienced insecurity about the procedure, especially if they had never tested themselves before.

It was probably a bit overwhelming, maybe because it was the first time. I just had to think about how to do it... not because it was difficult; I just had to figure it out

7

Thus, security was experienced when the instructions were balanced between being detail-oriented while also being simple and illustrative.

For some, a phone conversation with an HCP before the self-test created a sense of security because they could ask questions and receive information that “prepared them” for taking the test. However, anonymity was challenged for others due to the phone call with an HCP.

### Reducing Intrusive Feelings

Self-testing was experienced as an anonymous and discreet solution.

If you reduce the fear one may have, I believe it will make a difference. I easily believe that self-testing will encourage more people to get tested

7

The anonymity and discretion contributed to preserving privacy and removed the fear of an awkward conversation or being judged by HCPs, thus reducing intrusive feelings.

Testing for STIs is associated with taboo and shame, and this experience was minimized when the test could be taken anonymously.

So I avoided the awkwardness that can occur and the judgment one can encounter in the eyes of healthcare professionals. It was super easy and straightforward; it was actually very nice to experience

4

Many preferred using the self-test rather than going to their general practitioner because they only saw their doctor for other medical issues. This may be explained by the fear of being judged for their sexual behavior or irresponsibility in relation to unsafe sex.

For some, it took courage to get tested for an STI, and this courage was empowered when the test could be done anonymously. The fact that the self-test took place in a discreet and less crowded location promoted anonymity and a sense of privacy.

It feels a bit more anonymous when you come down and just take an envelope and test yourself without having a slightly awkward conversation with a doctor

6

Discussing one’s sexual behavior was experienced as invasive and judgmental, which could lead to hesitation in getting tested. However, using the STT reduced these barriers, enhanced feelings of security, and, in that way, supported patient self-care.

## Discussion

### Principal Findings

The development of new technology will inevitably lead to changes elsewhere in the health care organization [22]. The co-design process that led to the development and implantation of the STT ensured that the solution was integrated into existing clinical workflows and that HCPs and patients supported it. These critical aspects must be addressed to ensure the adoption and implementation of new technologies for STI testing [23]. Khumalo et al [23] stated that if new technologies are implemented correctly, patients will be provided with autonomy and be empowered to take control of their sexual health because barriers toward testing, such as stigma, can be reduced. This is consistent with the findings of our study, where being tested for STIs was associated with taboo and shame. However, taking an anonymous test minimized this experience, enhancing feelings of security.

Self-testing was seen as more appealing than a face-to-face consultation because it was an easy solution that both saved time and allowed for freedom and flexibility to plan the visit independently. These aspects highlight the improvements in offering testing using the STT compared with existing home test kits. The STT gives patients the opportunity to be tested quickly and enables them to get a test result within 1 to 2 days, which is considered an important aspect of STI testing. [7]. Aicken et al [24] found similar findings in their study of a newly established e-Sexual health clinic. The participants in their study described that they chose to use the eHealth intervention because it was convenient and fit into their busy lifestyle, and it was considered both easy and discreet to use. Likewise, helpline contact was considered important and created a sense of security for the majority of patients; however, like in our study, anonymity was challenged for some due to a phone call with an HCP. This highlights the extent to which STI testing is connected to feelings of shame and stigma and, therefore, the need to constantly improve and develop the services for this group using co-design approaches in order to facilitate patient self-care.

Orem defines self-care as activities an individual initiates and performs on their own behalf to maintain life, health, and well-being [25]. This involves adaptions to health-related behavior and the ability to perform self-care activities, referred to as self-care agencies. Thus, nursing has to support or enhance the individual’s self-care agency in order to promote independence [26]. We found that using the STT was perceived as orderly and meaningful for its purpose, which could create motivation for self-care by getting tested, which enhanced the possibility of taking responsibility for one’s own sexual health. This highlights a certain paradox: the ability to perform self-care depends not solely on the individual but also on a health care system that has to adapt to users. The routines in clinical practice, such as only providing face-to-face consultations despite the well-documented barriers this creates, can lead to self-care deficits, not caused by the individual but by the system. Thus, providing patients with the appropriate intervention to address self-care deficits is not only the responsibility of individual patients and HCPs but also the responsibility of the health care system. This study found that the STT solution provided patients with feelings of anonymity and allowed them to take the test on their own terms. This empowered patients because it gave them control and the ability to make choices, enhancing their confidence and feelings of self-determination. Furthermore, it underlines the possible impact technologies can have, such as enabling self-care for patients who may not be regarded as having self-care deficits. In that way, technology plays a crucial role in empowering patients in STI testing by providing them with tools and resources to take control of their health and well-being. By leveraging technology in these ways, health care systems can empower patients to be more proactive, engaged, and informed in managing their health and well-being. This, in turn, contributes to improved patient outcomes and a more patient-centered approach to health care, ultimately contributing to the prevention and early detection of STIs.

The STT described here is the first and only one of its kind used in STI testing in Denmark, and with the health care system under pressure, there is a need to explore new paths and seek innovative solutions. Furthermore, by addressing one of the significant threats to the health care system, the workforce shortage, this solution has increased patient satisfaction. It is important to have a range of different options and solutions to meet patients’ needs, and the use of technology and self-testing can seamlessly coexist as an offering alongside more traditional consultation, as demonstrated by this study. While some patients easily adapt to new solutions, such as self-testing, others may need assistance. It is important that support, such as informational videos and helpline numbers, is integrated to ensure accessibility and understanding.

Considering the digital divide and health equality, it is important to ensure accessibility of the STT for diverse populations, including those with limited access to technology or low eHealth literacy. This has been an important focus area in the process of designing the STT and the STT provides the possibility to support a more traditional way of providing information and health care more flexibly. For instance, the solutions allow to provide information in different ways (videos, text, and personal information), to make sure that all needs are met. It has been an important focus area to make sure that a health care professional can be contacted for any need of support with respect to the STT and to allow patients to be tested without using the STT, but instead attending a face-to-face consultation if preferred.

### Limitations

A limitation of this study is that it was single-centered and included only 13 patients in the feasibility study and 10 patients in the qualitative interview, which is a rather small sample size. However, this was a design and development process and an evaluation of technology to explore the experiences and perceptions of patients and the technology’s adaptability in clinical practice. This favors a qualitative approach, and thus, the sample size seems adequate since qualitative research is concerned with deepening the understanding of a phenomenon rather than numerical representability [27]. The qualitative approach was used to obtain an in-depth insight suitable to the aim and considered a main strength of this study. In addition, we aimed for maximum variation during recruitment, which is considered a strength [28]. In the qualitative evaluation study, we recruited heterosexual patients only and did not collect data about other risk factors; this could have an impact on the transferability of the findings. However, heterosexual patients comprise the main target group of those being tested at the sexual health clinic and, thus, contribute to a representative group. We acknowledge that STI testing intersects with various cultural beliefs and practices related to sexual health, influenced by social norms, religious teaching, sexual preferences, and stigma. In some cultures, discussing sexual health openly is considered taboo, leading to reluctance in being tested. In addition, barriers such as privacy concerns, fear of judgment, and accessibility may be some obstacles. The STT gives individuals the opportunity to get tested more anonymously, thus, circumventing potential barriers. However, the inclusion criteria for this study were heterosexual Danish-speaking participants, therefore the cultural aspects and how the STT accommodates diverse beliefs and practices related to sexual health needs to be investigated further.

In the feasibility study, we included patients who attended a face-to-face consultation and asked them if they would use the STT instead and that data would be collected through participant observation and a subsequent interview. We deliberately omitted to gather demographic data because the time to think over to agree to participate was sparse. This information may have strengthened the generalizability of the study; however, we chose to uphold research ethics [29]. Another limitation is that the perceptions and experiences of HCPs were not elaborated on, although the STT created a significant change in clinical practice. However, the STT was implemented quickly into clinical practice at the request of HCPs, which indicated that the STT was a demanded solution.

### Future Perspectives

It would be interesting to evaluate the use of the STT over a longer time period, monitoring the number of users, their sex and age, whether they had used the STT before, as well as the number of positive samples. Our clinic has plans to further automate the STT. This will eliminate the need for a telephone conversation with an HCP and enhance users’ anonymity. In addition, the STT should be expanded to users with a higher risk of STI infection, for example, pre-exposure prophylaxis users who are routinely tested for STIs regularly.

### Conclusions

Based on PD, we have designed and developed an STT that allows patients to be tested at a sexual health clinic through self-collected sampling without a face-to-face consultation. Using the STT minimized feelings of shame and awkwardness, which is a well-known barrier to STI testing and can contribute to a greater willingness to live with STIs. Thus, accessible health care services are crucial in preventing and reducing the impact of STIs, and the SST may increase testing uptake as it takes into account some of the barriers that exist. More simplified and accessible chlamydia testing by the STT proved feasible. The feasibility study and qualitative evaluation have resulted in a fully functioning implementation of the STT in clinical practice.

## Abbreviations

COREQ: Consolidated Criteria for Reporting Qualitative Studies
HCP: health care professional
PD: participatory design
STI: sexually transmitted infection
STT: self-testing technology

## References

[1] (2022). Statens Serum Institut. Klamydia - opgørelse over sygdomsforekomst 2019-2021.

[2] Hoenderboom BM, van Oeffelen AAM, van Benthem BHB, van Bergen JEAM, Dukers-Muijrers NHTM, Götz H M, Hoebe CJPA, Hogewoning AA, van der Klis FRM, van Baarle D, Land JA, van der Sande MAB, van Veen MG, de Vries F, Morré S A, van den Broek IVF (2017). The Netherlands Chlamydia Cohort Study (NECCST) protocol to assess the risk of late complications following chlamydia trachomatis infection in women. BMC Infect Dis.

[3] Davies B, Turner KME, Frølund M, Ward H, May MT, Rasmussen S, Benfield T, Westh H (2016). Risk of reproductive complications following chlamydia testing: a population-based retrospective cohort study in Denmark. Lancet Infect Dis.

[4] Balfe M, Brugha R (2010). Disclosure of STI testing activities by young adults: the influence of emotions and social networks. Sociol Health Illn.

[5] Gilbert M, Thomson K, Salway T, Haag D, Grennan T, Fairley CK, Buchner C, Krajden M, Kendall P, Shoveller J, Ogilvie G (2018). Differences in experiences of barriers to STI testing between clients of the internet-based diagnostic testing service GetCheckedOnline.com and an STI clinic in Vancouver, Canada. Sex Transm Infect.

[6] Trettin B, Vestergaard T, Stensgaard A (2015). Understanding young people’s barriers to sexually transmitted disease screening and meeting their needs: A focus group study. JNEP.

[7] Handy P, Pattman RS, Richards J (2006). 'I'm OK?' Evaluation of a new walk-in quick-check clinic. Int J STD AIDS.

[8] Grandahl M, Larsson M, Herrmann B (2020). 'To be on the safe side': a qualitative study regarding users' beliefs and experiences of internet-based self-sampling for Chlamydia trachomatis and Neisseria gonorrhoeae testing. BMJ Open.

[9] Clemensen J, Rothmann MJ, Smith AC, Caffery LJ, Danbjorg DB (2017). Participatory design methods in telemedicine research. J Telemed Telecare.

[10] Simonsen J, Robertson T, NA NA (2012). Routledge International Handbook of Participatory Design.

[11] Theunissen KATM, Bos AER, Hoebe CJPA, Kok G, Vluggen S, Crutzen R, Dukers-Muijrers NHTM (2015). Chlamydia trachomatis testing among young people: what is the role of stigma?. BMC Public Health.

[12] McDonagh LK, Saunders JM, Cassell J, Curtis T, Bastaki H, Hartney T, Rait G (2018). Application of the COM-B model to barriers and facilitators to chlamydia testing in general practice for young people and primary care practitioners: a systematic review. Implement Sci.

[13] Lee AS, Cody SL (2020). The stigma of sexually transmitted infections. Nurs Clin North Am.

[14] Balfe M, Brugha R, O'Donovan D, O'Connell E, Vaughan D (2010). Young women's decisions to accept chlamydia screening: influences of stigma and doctor-patient interactions. BMC Public Health.

[15] Spradley J, NA NA (2016). Participant Observation.

[16] Kvale S, Brinkmann S, NA NA (2009). Learning the Craft of Qualitative Research Interviewing.

[17] World Medical Association (2013). World Medical Association Declaration of Helsinki: ethical principles for medical research involving human subjects. JAMA.

[18] Moltu C, Stefansen J, Svisdahl M, Veseth M (2012). Negotiating the coresearcher mandate - service users' experiences of doing collaborative research on mental health. Disabil Rehabil.

[19] Ricoeur P (1976). Interpretation Theory: Discourse and the Surplus of Meaning.

[20] Simonÿ C, Specht K, Andersen IC, Johansen KK, Nielsen C, Agerskov H (2018). A Ricoeur-inspired approach to interpret participant observations and interviews. Glob Qual Nurs Res.

[21] Tong A, Sainsbury P, Craig J (2007). Consolidated criteria for reporting qualitative research (COREQ): a 32-item checklist for interviews and focus groups. Int J Qual Health Care.

[22] Leavitt H (2013). Applied Organizational Change in Industry: Structural, Technological and Humanistic Approaches.

[23] Khumalo F, Passmore J, Manhanzva M, Meyer B, Duyver M, Lurie M, Tanko RF, Masson L (2023). Shifting the power: scale-up of access to point-of-care and self-testing for sexually transmitted infections in low-income and middle-income settings. Curr Opin Infect Dis.

[24] Aicken CRH, Sutcliffe LJ, Gibbs J, Tickle LJ, Hone K, Harding-Esch EM, Mercer CH, Sonnenberg P, Sadiq ST, Estcourt CS, Shahmanesh M (2018). Using the eSexual health clinic to access chlamydia treatment and care via the internet: a qualitative interview study. Sex Transm Infect.

[25] Denyes MJ, Orem DE, Bekel G (2001). Self-care: a foundational science. Nurs Sci Q.

[26] Orem DE, Calnan ME, Renpenning KM (1995). Nursing: Concepts of Practice.

[27] Queirós A, Faria D, Almeida F (2017). Strengths and limitations of qualitative and quantitative research methods. European Journal of Education Studies.

[28] Polit D, Beck C (2010). Essentials of Nursing Research: Appraising Evidence for Nursing Practice.

[29] Damianakis T, Woodford MR (2012). Qualitative research with small connected communities: generating new knowledge while upholding research ethics. Qual Health Res.

